# A machine learning framework to determine geolocations from metagenomic profiling

**DOI:** 10.1186/s13062-020-00278-z

**Published:** 2020-11-23

**Authors:** Lihong Huang, Canqiang Xu, Wenxian Yang, Rongshan Yu

**Affiliations:** 1 School of Informatics, Xiamen University, Xiamen, China; 2 Aginome Scientific Pte. Ltd., Xiamen, China

**Keywords:** Abundance profiling, Binning, Affine transform, Kriging interpolation

## Abstract

**Background:**

Studies on metagenomic data of environmental microbial samples found that microbial communities seem to be geolocation-specific, and the microbiome abundance profile can be a differentiating feature to identify samples’ geolocations. In this paper, we present a machine learning framework to determine the geolocations from metagenomics profiling of microbial samples.

**Results:**

Our method was applied to the multi-source microbiome data from MetaSUB (The Metagenomics and Metadesign of Subways and Urban Biomes) International Consortium for the CAMDA 2019 Metagenomic Forensics Challenge (the Challenge). The goal of the Challenge is to predict the geographical origins of mystery samples by constructing microbiome fingerprints.First, we extracted features from metagenomic abundance profiles. We then randomly split the training data into training and validation sets and trained the prediction models on the training set. Prediction performance was evaluated on the validation set. By using logistic regression with L2 normalization, the prediction accuracy of the model reaches 86%, averaged over 100 random splits of training and validation datasets.The testing data consists of samples from cities that do not occur in the training data. To predict the “mystery” cities that are not sampled before for the testing data, we first defined biological coordinates for sampled cities based on the similarity of microbial samples from them. Then we performed affine transform on the map such that the distance between cities measures their biological difference rather than geographical distance. After that, we derived the probabilities of a given testing sample from unsampled cities based on its predicted probabilities on sampled cities using Kriging interpolation. Results show that this method can successfully assign high probabilities to the true cities-of-origin of testing samples.

**Conclusion:**

Our framework shows good performance in predicting the geographic origin of metagenomic samples for cities where training data are available. Furthermore, we demonstrate the potential of the proposed method to predict metagenomic samples’ geolocations for samples from locations that are not in the training dataset.

**Supplementary Information:**

The online version contains supplementary material available at (doi:10.1186/s13062-020-00278-z).

## Background

The advance of Next Generation Sequencing (NGS) technologies has made it possible to generate metagenomic sequencing data from microbiome samples at reasonable costs. The vast amount of sequencing data and the modern machine learning tools for data analytics are driving the advances in microbial ecology. Of the world’s 7.4 billion people, more than half (57%) live in urban areas (http://wdi.worldbank.org/table/3.12). With approximately 30% of urban populations using subways and buses, public transportation becomes one of the most shared spaces of the urban environment. Within this shared space, there exists a rich and diverse world of unseen organisms in constant interaction with human. The MetaSUB Consortium studies genetic interactions and community compositions of mass-transit biomes through DNA sequencing and computational modeling. It is well-known that environmental microbial communities differ in composition and functions across different geographical locations, possibly due to variations in climate, rainfall, altitude, soil, as well as the metabolic properties of their host or energy sources available in the environment. The MetaSUB Consortium is aiming at improving city utilization and planning through the detection, measurement, and design of metagenomics within urban environments [1]. Furthermore, the study of environmental metagenomics also brings potentials in new drug development as well as forensic applications such as identification of geographical origins of environmental samples.

In this paper, we focus on the prediction of geolocation, or city-of-origin, of a given metagenomic sample based on a training dataset that contains microbial samples from a set of different cities. We investigate the prediction problem under two separate but interconnected scenarios. In the first scenario, the city-of-origin of the given sample occurs in the training dataset, i.e., the city has been sampled before and the features or biological fingerprints of the city have been learnt. The geolocation prediction problem in this scenario closely resembles a classical multi-class classification problem with readily available solutions from machine learning. In the second scenario, the metagenomic sample may come from a city that has not been sampled before (unsampled city), which is a more unusual setting for machine learning. In such a case, we first use the prediction model from the first scenario to calculate the probabilities of the samples from cities in the training set (sampled city). The predicted probabilities were then used as anchor points to interpolate the probabilities of the sample from unsampled cities based on the continuity assumption on the geographic distribution of microbial samples on the map.

Previous works on geolocation prediction of metagenomic samples in the first problem can be found in [2–5]. Geolocation prediction in those works typically involves a feature extraction step and a modeling step that performs training and prediction on the extracted features. As the number of training samples can be very limited comparing to the large diversity of bacteria strains in each sample, the prediction problem in this scenario is a typical “large *p*, small *n*” problem with a few data points and many features. Therefore, feature extraction is a critical step to avoid the potential overfitting problem that deteriorates the location prediction performance on testing data. It is intuitive to use bacteria abundance profiles extracted from the metagenomic data for geolocation prediction. Many metagenomic profiling tools can be used for this purpose, and some of these tools are reviewed and evaluated in [6]. Further processing of raw features mainly targets at dimension reduction by extracting features with functional significance and differentiating power. In [2], differentially abundant bacteria are selected as features. In [3], sequence reads are classified into known taxonomic groups and per read counts of each taxonomic rank per sample are used as raw features. In [4], functional profiles, instead of abundance profiles, are used as features, as their biological interpretation is more straightforward. Feature downsampling or dimension reduction can be done by principal component analysis (PCA), Robust PCA [2], t-distributed Stochastic Neighbor Embedding (t-SNE), etc. Once features are extracted, the prediction problem can be readily solved using a pool of machine learning tools, e.g., *k*-NN [7], random forest [8], logistic regression [9], XGBoost [10], multi-layer perceptrons (MLP) [11], etc.

To determine the geolocation in the second scenario is more challenging as machine learning algorithms can only assign the probabilities of a testing sample to the cities in the training set. Relatively fewer works have been done in this scenario. In [4], testing datasets with 3 unsampled cities from CAMDA 2018 (http://camda2018.camda.info/) were used to evaluate the similarity of microbial samples between cities using bacteria abundance as features. Results show the tendency that samples cluster on the feature space based on their geographic locations, but outliers exist. The results suggest that geographical distance is an important factor, and it may be possible to infer cities-of-origin of samples based on their geographical distances to the cities in the training set. However, the geographical distance may not accurately reflect the biological distance between samples from different cities by itself. Hence, further modifications are necessary to mitigate the gap between geographical and biological distances for more reliable prediction results.

## Methods

### Data

We downloaded data from the CAMDA 2019 Metagenomics Forensic Challenge (http://camda2019.camda.info/) provided by CAMDA in partnership with MetaSUB International Consortium. The training dataset composes of 305 samples from 16 cities (Auckland, Berlin, Bogota, Hamilton, Hong Kong, Ilorin, London, Marseille, New York, Offa, Porto, Sacramento, Sao Paulo, Sofia, Stockholm, Tokyo) with unbalanced distribution, and the testing dataset contains 61 mystery samples from unsampled cities that do not occur in the training dataset. These datasets provide a unique resource for the study of biodiversity within and across geographic locations for metagenomic forensics. All data were Illumina-sequenced at variable depth values and were provided in compressed FASTQ format with DSRC tool [12]. Detail of the datasets is included in Table 1.

**Table 1:** MetaSUB metagenomic dataset description

Set	City	Country	Sample count
Training Set	Auckland (AKL)	New Zealand	14
	Berlin (BER)	Germany	21
	Bogota (BOG)	Colombia	15
	Hamilton (HAM)	New Zealand	16
	Hong Kong (HGK)	China	18
	Ilorin (ILR)	Nigeria	24
	London (LON)	U.K.	24
	Marseille (MAR)	France	10
	New York (NYC)	U.S.A.	26
	Offa (OFA)	Nigeria	20
	Porto (PXO)	Portugal	20
	Sacramento (SAC)	U.S.A.	18
	Sao Paulo (SAO)	Brazil	24
	Sofia (SOF)	Bulgaria	10
	Stockholm (STO)	Sweden	20
	Tokyo (TOK)	Japan	25
Total size	779 Gb		
Testing Set	Rio de Janerio	Brazil	12
	Santiago de Chile	Chile	6
	Kiev	Ukraine	8
	Brisbane	Australia	7
	Vienna	Austria	5
	Doha	Qatar	3
	Pairs	France	8
	Oslo	Norway	12
Total size	219 Gb		

### Data preprocess

First of all, the raw metagenomic sequencing data were preprocessed by fastp (v.0.19.4; https://github.com/OpenGene/fastp) [13] for quality control including automatic filtering, trimming, and error removing. For all samples used in our experiments, we trimmed the front of both reads in a pair (for paired-end reads) or the single read (for single-end reads) with options (-f 15 -F 15), and performed per-read cutting-by-quality in the tail (--cut_tail).

After quality control, the reads are filtered against human reference genome to remove possible contamination. First, the reads are aligned to human reference genome hg19 [14] using bwa mem (v.0.7.12-r1039; https://github.com/lh3/bwa.git) [15] with options (-t 44 -L 200), and the aligned SAM is filtered using samtools view (v.1.3.1; https://github.com/samtools/samtools.git) [16] with options (-@44 -F 2316). In this way, we filtered out the reads with exact match to human reference genome, and the retained reads were used to calculate the taxonomic abundance profile.

### Feature extraction

For each sample, we estimated its clade-abundance using MetaPhlAn2 (v.2.7.5; http://huttenhower.sph.harvard.edu/metaphlan) [17] and Kraken2 (v.2.0.8-beta; https://ccb.jhu.edu/software/kraken2/) [18]. Each taxonomic classification tool provides the relative abundance of all detected clades in the microbiome sample as percentages from kingdom level to species level. We used all taxonomic level abundances as our starting point for further feature selection. Since not all taxonomies are present in all cities, missing taxonomies were filled with zero so that all cities have an equal number of features.

Note that the number of samples per city varies between 10 and 26 (Table 1). Due to the scarcity of training samples, we used the following methods to avoid potential overfitting in training the prediction model.

First, we discretized the abundance profile data by binning the abundance profiles to ternary values {-1,0,1}. Data discretization can prevent the prediction model from predicting the target using trivial small variations in the input feature vectors. The binning operation is based on the sample-wise percentile of the abundance profiles of all training samples. Particularly, we used the following equation

1 $$  f(x)=\left\{\begin{array}{ll} -1, & \text{if}\,\, x<P25\\ 0, & \text{if}\,\, P25\leq x<P75 \\ 1, & \text{if}\,\, x\geq P75 \end{array}\right.  $$

to discretize the abundance profile, where *P*25 and *P*75 represent the 25*th* and 75*th* percentile, respectively. In this way, only salient differences among samples from different cities could be recognized as features in training.

Secondly, we applied Recursive Feature Elimination (RFE) to select a subset of features that provides good differentiation power for target identification from the original abundance profile. RFE selects features by repeatedly constructing prediction models with decreasing number of features, and then choose the best model based on the prediction performance on the validation set. In this work, we used the REF function provided by the scikit-learn package of Python with step size 5 (*step*=5) to perform RFE [19].

### Model training

With the extracted features from the abundance profiles, the problem of determining the geolocation of a sample can be solved as a multi-class classification problem if the testing sample is from a sampled city in the training set.

In our framework, we trained a logistic regression model with L2 regularization using the one-vs.-rest approach to produce a multi-class classifier. For a test sample, the trained model will produce 16 probability values, indicating the possibility of this sample coming from each of the 16 cities, respectively. For each sampled city, the output from the logistic prediction model can be represented as:

2 $$  \vec{p}=\frac{1}{1+e^{-\left(\mathbf{w}^{T}\vec{x}+\vec{\theta}\right)}},  $$

where $\vec {x}$ denotes the input features extracted from the sample, $\vec {p}$ denotes the output probability of the sample on sampled cities, **w** and $\vec {\theta }$ are learnt model parameters.

We also tested tree-based classification algorithms that natively support multi-class classification, such as XGBoost and Random Forest. Their performance is very close to the logistic regression algorithm on the provided dataset.

### Kriging interpolation

In some cases, people may be interested to check the probability of a testing sample from a city not sampled before if they know a priori that the city-of-origin of that sample is not included in the training set. The multi-class classifier only gives the probabilities of a testing sample from sampled cities. To derive its probabilities from unsampled cities, we propose to interpolate from predicted probabilities on sampled cities under a continuity assumption on the distribution of abundance profile on the map. For this purpose, we used Kriging interpolation [20] originated from geostatistics to estimate the “fill-in” probabilities of spatial locations between sampled cities. Kriging interpolation produces the optimal linear unbiased prediction of intermediate values under the assumption of wide sense stationary of covariance on the map. Using Kriging interpolation, the probability $\hat {p}_{o}$ that a sample is from an unsampled city at coordinate (*x*_*o*_,*y*_*o*_) is given by:

3 $$  \hat{p}_{o}=\sum_{i=1}^{n}\lambda_{o,i}\cdot p_{i}  $$

where *p*_*i*_ denotes the predicted probability that the sample is from city *i*, and *λ*_*o*,*i*_ denotes the weight for city *i* calculated w.r.t. target coordinate (*x*_*o*_,*y*_*o*_). *λ*_*o*,*i*_ can be obtained by solving the following contrained optimzation problem

4 $$ \lambda_{o,i}=\underset{\lambda}{\min}\,\, E\left(\hat{p}_{o} - p_{o}\right)^{2},  $$

subject to

5 $$ E\left(\hat{p}_{o} - p_{o}\right) = 0.  $$

Here, *E*(·) denotes statistical expectation. The detailed mathematical derivation of the solutions can be found in [21], and we used the PyKriging toolbox (http://pykriging.com) in our implementation.

### Biological coordinate system

By using Kriging interpolation to determine the probabilities of testing samples from unsampled cities, we assumed that the spatial distribution of abundance profile from different cities follows Tobler’s First Law of Geography, i.e., “everything is related to everything else, but near things are more related than distant things” [22]. However, this assumption may not always be valid. In some cases, cities that are geographically further away may have more similar abundance profiles, comparing to cities that are closer [4]. To overcome this limitation, we defined a biological coordinate system on which the distance between cities better reflects their similarity in terms of biological differences.

The biological coordinate system was derived as follows. First, we performed PCA on the abundance profiles of all samples from the training set, which gave us a 2D vector $\left (\vec {x}_{i},\vec {y}_{i}\right)$ for each sample. We then calculated the centroid of all samples from city *c* as the biological coordinates of city *c*, i.e.,

6 $$  \left\{\begin{array}{ll} x_{c} = {\sum\nolimits}_{i=1}^{n} x^{c}_{i} / {n}, \\ y_{c} = {\sum\nolimits}_{i=1}^{n} y^{c}_{i} / {n}, \end{array}\right.  $$

where *n* is the total number of samples from city *c* in the training set, and $\left (x^{c}_{i},y^{c}_{i}\right), (i=1,\ldots,n)$ denotes their locations in the biological coordinate system.

Finally, based on the biological coordinates of all sampled cities, the biological coordinates of unsampled cities can be derived by applying affine transformation [23] between the biological and geographical coordinate systems, using the coordinates of sampled cities as anchor points as follows:

7 $$ \left[\begin{array}{ccc} x& y& 1 \end{array}\right] \times \left[\begin{array}{cccc} m_{11} & m_{12} & 0 \\ m_{21} & m_{22} & 0 \\ t_{x} & t_{y} & 1 \end{array}\right] = \left[\begin{array}{cccc} x^{'} & y^{'} & 1 \end{array}\right].  $$

**Fig. 1:**
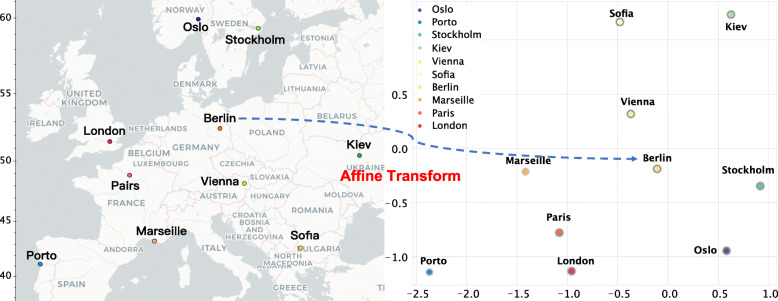
Testing samples were geotagged with longitude and latitude coordinates via global positioning system (GPS). After that, we used affine transformation to transform geographic points to biological points, and applied Kriging interpolation to predict the probabilities of the testing samples from unsampled cities

**Fig. 2:**
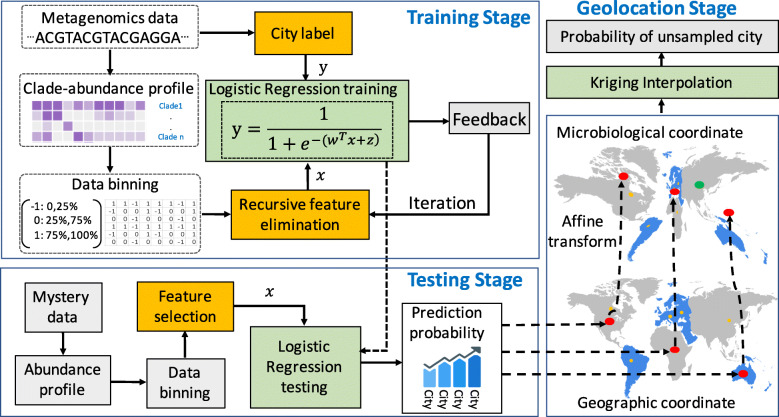
The flowchart of the proposed framework

Here, (*x*,*y*) and $\phantom {\dot {i}\!}\left (x^{'}, y^{'}\right)$ are, respectively, the locations of a city in its geographical and biological coordinates, and *m*’s and *t*’s are the affine transform parameters obtained from the anchor points based on least squares fit [24]. Kriging interpolation was further performed on the biological coordinate system to derive the probabilities of the testing samples on unsampled cities (Fig. 1). The flowchart of the proposed framework is in Fig. 2.

## Results and discussion

### Feature selection

Data preprocessing is applied to all training samples as described in “Methods” section. We generated 2762 raw abundance profiles using MetaPhlAn2 and 5503 raw abundance profiles using Kraken2. We then performed a binning operation on the raw abundance profiles. We compared the prediction performance based on MethPhlAn2- and Kraken2-derived features with 100 times random shuffle-split of training and validation sets on the provided data. In each random split, RFE was performed on the training dataset to select most important features, and the performance was evaluated based on the average accuracy on the validation set.

**Fig. 3:**
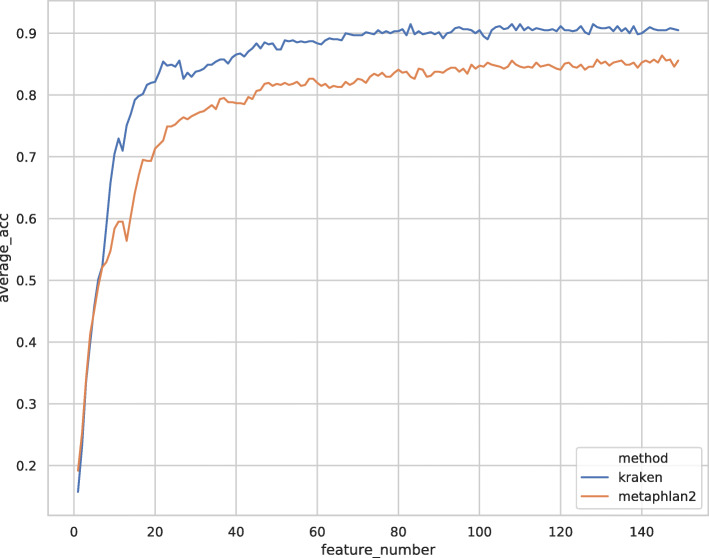
Prediction performance on validation set v.s. number of features used for training on the corresponding training data. Prediction performance is measured by averaging the prediction accuracy over 1000 random shuffle-splits of training and validation sets on the training dataset provided by the Challenge

We observed that classification performance increases with an increasing number of selected features from RFE (Fig. 3). However, the rate of performance increment slows down when the number of features exceeds 50. Therefore, we limited the number of features to 50 to prevent overfitting. In addition, as the average accuracy performance when using Kraken2-derived features is slightly better than that when using MetaPhlAn2-derived features, Kraken2-derived features were used in the subsequent experiments.

**Fig. 4:**
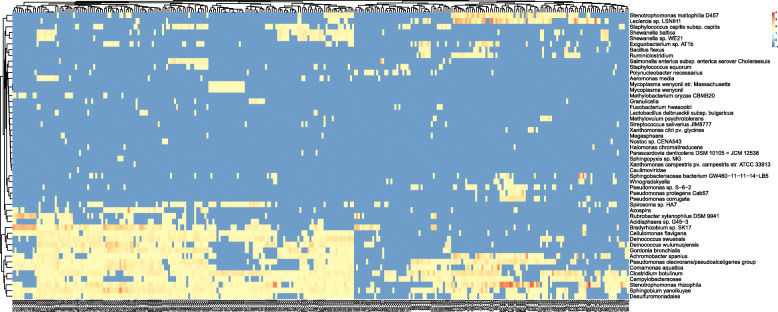
Two-dimensional hierarchical clustering of abundance profiles on the 50 selected features for all training samples. Abundance profiles are shown after log10(value+1e-6) operation

**Fig. 5:**
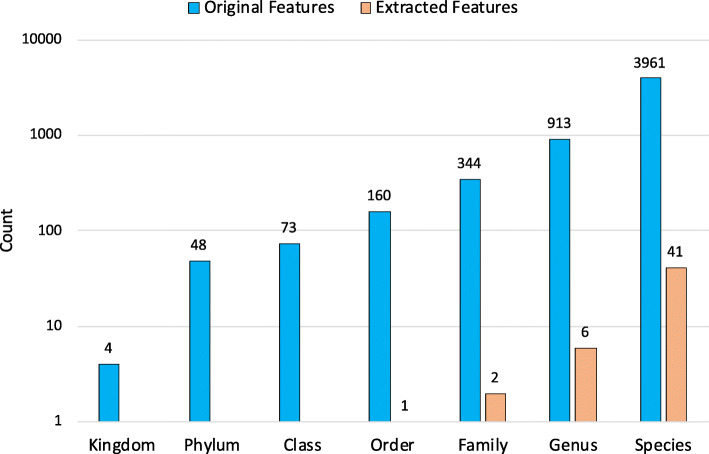
The total number of raw features is 5503, including 4, 48, 73, 160, 344, 913 and 3961 for Kingdom, Phylum, Class, Order, Family, Genus and Species clade levels, respectively. After feature selection, we retained 4 clade levels including Order, Family, Genus and Species. Species has the highest number of features (41) and Order has the lowest number of features (1)

We performed unsupervised clustering of samples in the data based on the top 50 Kraken2 features derived using RFE. In general, samples from one city cluster into one distinct group, and different cities like Sacramento, Offa, Tokyo, Stockholm, New York, form separate groups with distinct species patterns (Fig. 4). In addition, we found that features at species level play a significant role in the selected features (41 out of 50 features are at species level; see Fig. 5). Nearly half of the selected features (38%) are human pathogens and bacterial phytopathogens enriched at species, including *Salmonella enterica subsp. enterica serovar Choleraesuis*, *Staphylococcus capitis subsp. capitis*, *Fusobacterium hwasookii*, *Bacillus flexus* and *Streptococcus salivarius*. Other prevalent bacterial clades are involved in carbon and nitrogen of metabolism, including *Lactobacillus delbrueckii subsp. bulgaricus*, *Rubrobacter xylanophilus DSM 9941*, *Cellulomonas flavigena* and *Comamonas aquatica*.

**Fig. 6:**
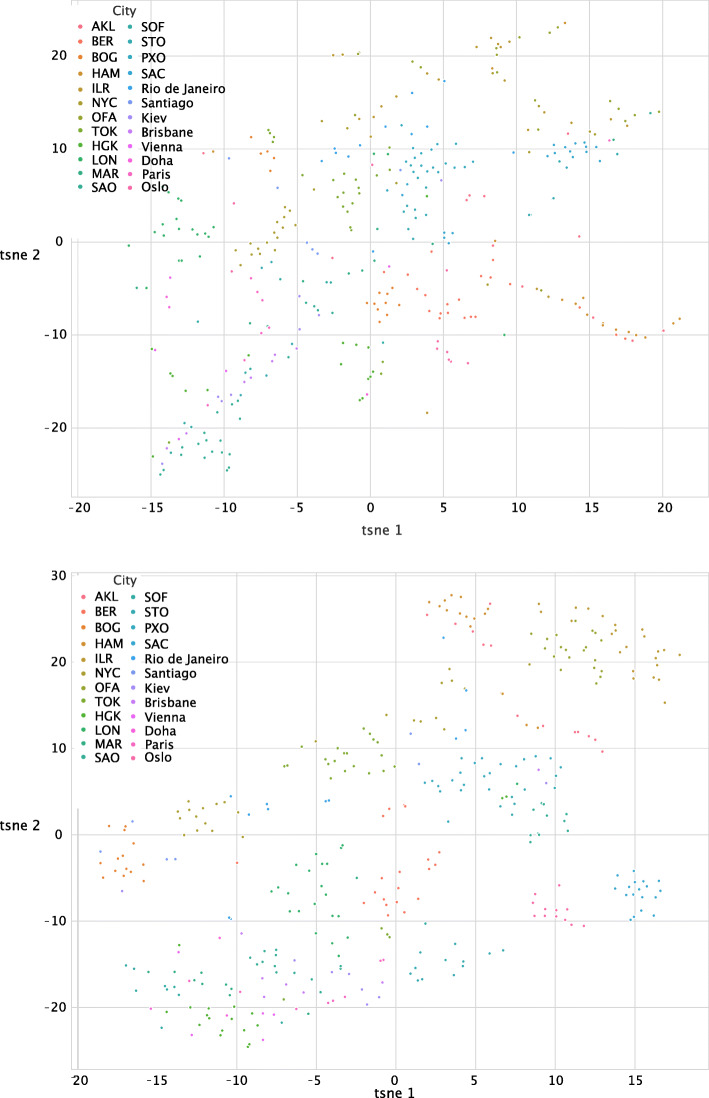
t-SNE visualization (**a**) before and (**b**) after feature binning. Before feature binning, it is very difficult to separate the cities. After binning, most cities can be clearly separated from others

**Fig. 7:**
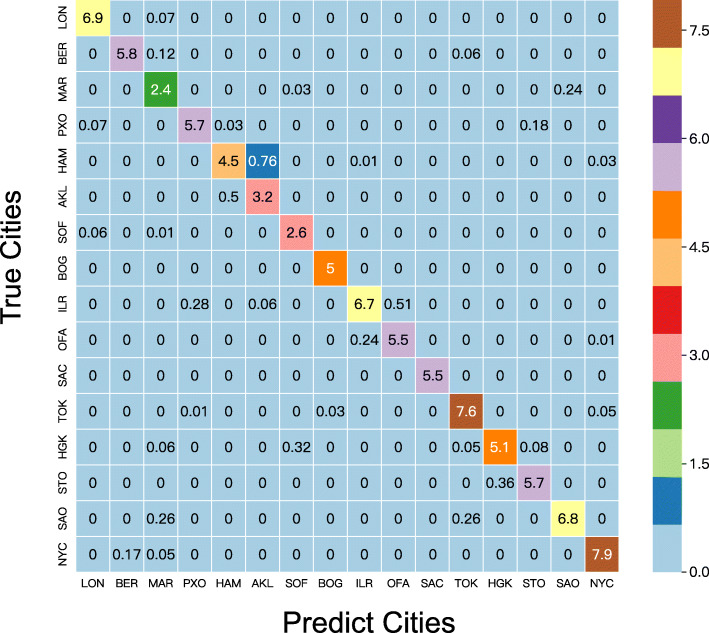
The confusion matrix of the training dataset calculated using binned abundance profiles. All cities are highly distinguishable except for Hamilton and Auckland. Both cities are well separated from the other cities, but it is relatively difficult to distinguish between these two cities

We also observed that when the same set of features were used, cities can be separated better with the binning operation. We visualized all the training samples based on the selected features using t-SNE (Fig. 6), which shows that the cities can be more clearly separated from others based on extracted features after feature binning. Further analysis from the confusion matrix shows that most cities are highly distinguishable except for two New Zealand cities, Hamilton and Auckland (Fig. 7), which are difficult to be separated although both cities, as a whole, can be separated from other cities.

### Geolocation prediction on sampled cities

We applied several widely used multi-class classification algorithms on the binned features to predict the probabilities of metagenomic samples belonging to cities from the training data. For quantitative evaluation, we performed *k*-fold stratified shuffle split for cross validation in model training with *k*=100. We used 70% of the training data for training and the remaining 30% for validation with random split. In each random split, the top 50 Kraken2 features derived from the training data were used as the prediction features. The average accuracy on validation sets reaches 86% by Logistic Regression, 81% by Random Forest [25], 83% by XGBoost and 80% by *k*-NN. Based on the performance, the final model was built using Logistic Regression classifier.

### Geolocation prediction on unsampled cities

To calculate the probability of a mystery sample from a city not sampled before, we performed Kriging interpolation based on its predicted probabilities on sampled cities. As the underlying principle behind Kriging interpolation assumes certain geographic proximity among observed values, we used only sampled cities from Europe for interpolation as other cities are scattering around the world. Similarly, we used only mystery samples from European cities in our evaluation. Since there are only 6 European cities in the training set which is far from sufficient for interpolation, we combined with all samples from the European cities in the provided testing dataset, resulting in 10 European cities in total. Then, for each city, we interpolated the probabilities of testing samples from this city based on their predicted probabilities on the other 9 European cities. Note that in predicting the probability of a given testing sample, only data from other cities were used in training the multi-class classifier and performing Kriging interpolation with affine transform. Therefore, there was no information leaking on the ground truth geolocation when individual samples were tested in our validation.

**Fig. 8:**
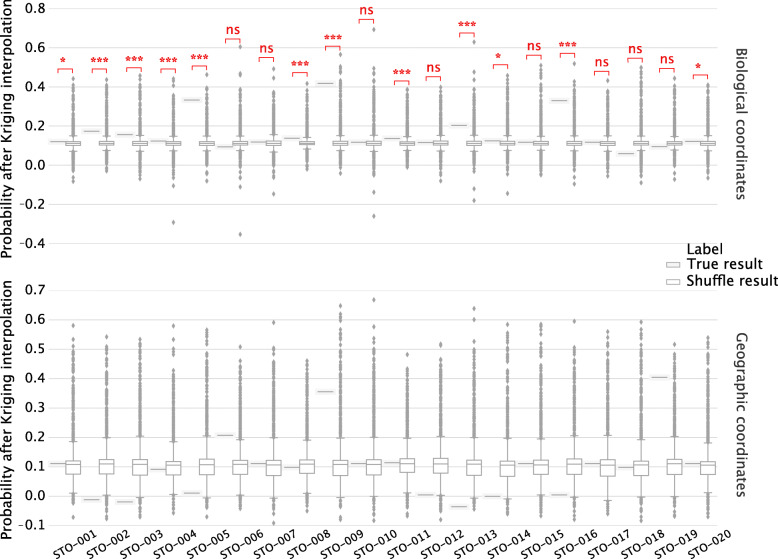
The probabilities were interpolated from the other 9 European cities except Stockholm using the proposed framework. Kriging interpolation is performed on the biological coordinates. For each sample, the probability on the left side is resulted from original training data and those on the right side are from permuted training samples. *P*-values for significant differences are noted

We observed that our geolocation prediction framework successfully assigned a certain number of samples to their cities-of-origin with high probabilities (illustrated in Fig. 8 using Stockholm as an example). To assess whether the high probabilities could have been generated by chance, we permuted the training samples 1000 times and performed our algorithm again on the permuted samples to regenerate the probabilities of the testing samples on their ground truth cities. It can be seen that for samples achieving high probabilities on their ground truth cities, the resulting distribution is significantly lower than those obtained from real training data (Fig. 8, SupplementaryFig. S1 to Fig. S9). Particularly, interpolation results from biological coordinates show much higher confidence compared to those from geographic coordinates, suggesting that biological coordinates could better reflect the deviation of the abundance profiles of metagenomic samples of different locations for inferring geolocation of unsampled sites.

**Fig. 9:**
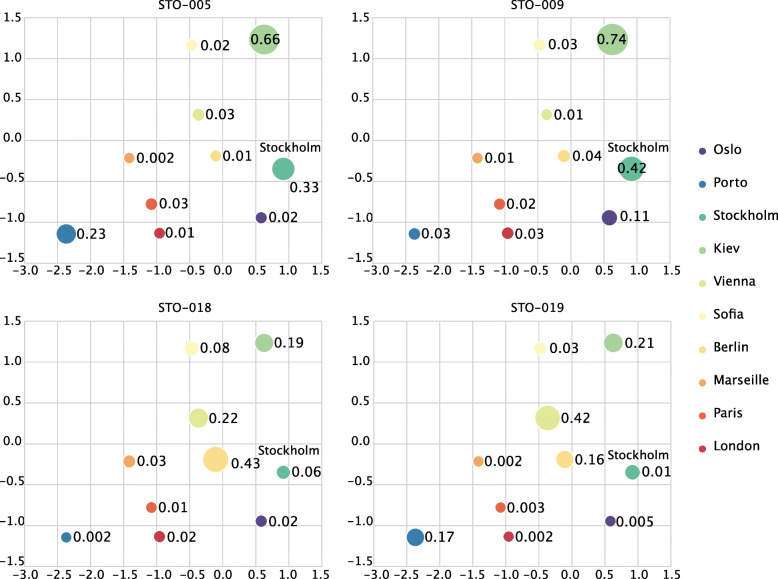
The predicted probabilities on nine cities from the training set and the interpolated probabilities of four samples from Stockholm shown on biological coordinate. The circle size indicates the probability

The results also show that the algorithm performs poorly on some testing samples, which could be possibly due to the limited number of cities from the training set compared to the size of the geographic coverage of those cities, as well as the small number of available training samples for each city, which may not cover all potential microbial from the city. To further illustrate this limitation, we showed the predicted and the interpolated probabilities of four different samples from Stockholm in Fig. 9. As can be seen from this figure, the probability of a sample from Stockholm will be largely determined by its predicted probabilities from cities nearby Stockholm in the biological coordinate, which is as expected. On the other hand, for samples that are not assigned with high probabilities to cities near Stockholm, the prediction performance would be poor since the interpolated probabilities will be low (STO-018, STO-019).

## Conclusion

In this work, we developed a framework using abundance profiles to predict the geolocation of metagenomic samples. The proposed method provides accurate predictions of geolocation of microbial samples using selected abundance profiles as features when the ground truth cities are sampled in the training dataset. For more challenging tasks where the ground truth cities are not sampled, the framework interpolates the probabilities of the testing samples from different cities based on their predicted probabilities on sampled cities. Note that although the interpolated probabilities may not be decisive as other surrounding cities may have the same or even higher probabilities than the city-of-origin, the derived probabilities are still useful for forensic applications where evidence from other sources could be combined to identify the true location under Bayesian framework. The effectiveness of the proposed framework was validated using the CAMDA 2019 dataset, while only partially due to limited available training data. It is envisioned that the performance of the proposed framework could be further validated once larger databases with more microbial samples and denser distribution of sampling cities become available in the future.

## Supplementary Information

**Additional file 1** Supplementary material for “A Machine Learning Framework to Determine Geolocations from Metagenomic Profiling”.

## Data Availability

The data used in this manuscript is available from CAMDA 2019 Metagenomic Forensics Challenge (http://camda2019.bioinf.jku.at/doku.php/contest_dataset). The code is publicly available at https://github.com/xmuyulab/metageopred.

## Abbreviations

CAMDA: Critical assessment of massive data analysis
MetaSUB: The metagenomics and metadesign of subways and urban biomes
NGS: Next generation sequencing
PCA: Principal component analysis
t-SNE: t-distributed stochastic neighbor embedding
XGBoost: Extreme gradient boosting
MLP: Multi-layer perceptrons
RFE: Recursive feature elimination

## References

[1] Consortium TMI (2016). The metagenomics and metadesign of the subways and urban biomes (metasub) international consortium inaugural meeting report. Microbiome.

[2] Alshawaqfeh M, Bashaireh A, Serpedin E, Suchodolski J (2017). Consistent metagenomic biomarker detection via robust PCA. Biol Direct.

[3] Ryan FJ (2019). Application of machine learning techniques for creating urban microbial fingerprints. Biol Direct.

[4] Casimiro-Soriguer CS, Loucera C, Perez Florido J, López-López D, Dopazo J (2019). Antibiotic resistance and metabolic profiles as functional biomarkers that accurately predict the geographic origin of city metagenomics samples. Biol Direct.

[5] Harris ZN, Dhungel E, Mosior M, Ahn T-H (2019). Massive metagenomic data analysis using abundance-based machine learning. Biol Direct.

[6] Zolfo M, Asnicar F, Manghi P, Pasolli E, Tett A, Segata N (2018). Profiling microbial strains in urban environments using metagenomic sequencing data. Biol Direct.

[7] Cover TM, Hart PE (1967). Nearest neighbor pattern classification. IEEE Trans Inf Theory.

[8] Breiman L (2001). Random forests. Mach Learn.

[9] Hosmer Jr DW, Lemeshow S, Sturdivant RX (2013). Applied logistic regression, vol. 398.

[10] Chen T, Guestrin C (2016). XGBoost: a scalable tree boosting system. Proceedings of the 22Nd ACM SIGKDD International Conference on Knowledge Discovery and Data Mining. KDD ’16.

[11] Du K-L, Swamy MNs (2014). Multilayer Perceptrons: Architecture and Error Backpropagation. Neural Networks and Statistical Learning.

[12] Roguski l., Deorowicz S (2014). DSRC 2 – industry-oriented compression of FASTQ files. Bioinformatics.

[13] Chen S, Zhou Y, Chen Y, Gu J (2018). fastp: an ultra-fast all-in-one FASTQ preprocessor. Bioinformatics.

[14] Venter JC, Adams MD, Myers EW, Li PW, Mural RJ, Sutton GG, Smith HO, Yandell M, Evans CA, Holt RA (2001). The sequence of the human genome. Science.

[15] Li H. Aligning sequence reads, clone sequences and assembly contigs with bwa-mem. arXiv preprint arXiv:1303.3997. 2013.

[16] Li H, Handsaker B, Wysoker A, Fennell T, Ruan J, Homer N, Marth G, Abecasis G, Durbin R (2009). The sequence alignment/map format and samtools. Bioinformatics.

[17] Truong DT, Franzosa EA, Tickle TL, Scholz M, Weingart G, Pasolli E, Tett A, Huttenhower C, Segata N (2015). MetaPhlAn2 for enhanced metagenomic taxonomic profiling. Nat Methods.

[18] Wood DE, Lu J, Langmead B. Improved metagenomic analysis with Kraken 2. bioRxiv,. 2019:762302. 10.1101/762302. 10.1186/s13059-019-1891-0 PMC6883579 31779668

[19] Guyon I, Weston J, Barnhill S, Vapnik V (2002). Gene selection for cancer classification using support vector machines. Mach Learn.

[20] Le ND, Zidek JV (2006). Statistical analysis of environmental space-time processes.

[21] Forrester A, Sobester A, Keane A (2008). Engineering design via surrogate modelling: a practical guide.

[22] Tobler WR (1970). A computer movie simulating urban growth in the detroit region. Econ Geogr.

[23] Berger M (1987). Geometry I.

[24] Späth H (2004). Fitting affine and orthogonal transformations between two sets of points. Math Commun.

[25] Walker AR, Datta S (2019). Identification of city specific important bacterial signature for the metasub camda challenge microbiome data. Biol Direct.

