# Supplementary material for: A machine learning framework to determine geolocations from metagenomic profiling
Source: Biol Direct. 2020 Nov 23;15:27. doi: 10.1186/s13062-020-00278-z (PMC7682025; doi:10.1186/s13062-020-00278-z)
Supplement: Supplementary file 1 — Additional file 1 Supplementary material for “A Machine Learning Framework to Determine Geolocations from Metagenomic Profiling”. [file 13062_2020_278_MOESM1_ESM.pdf]

Supplementary material for “A Machine Learning Framework to Determine Geolocations from Metagenomic Profiling”

L. Huang et al.

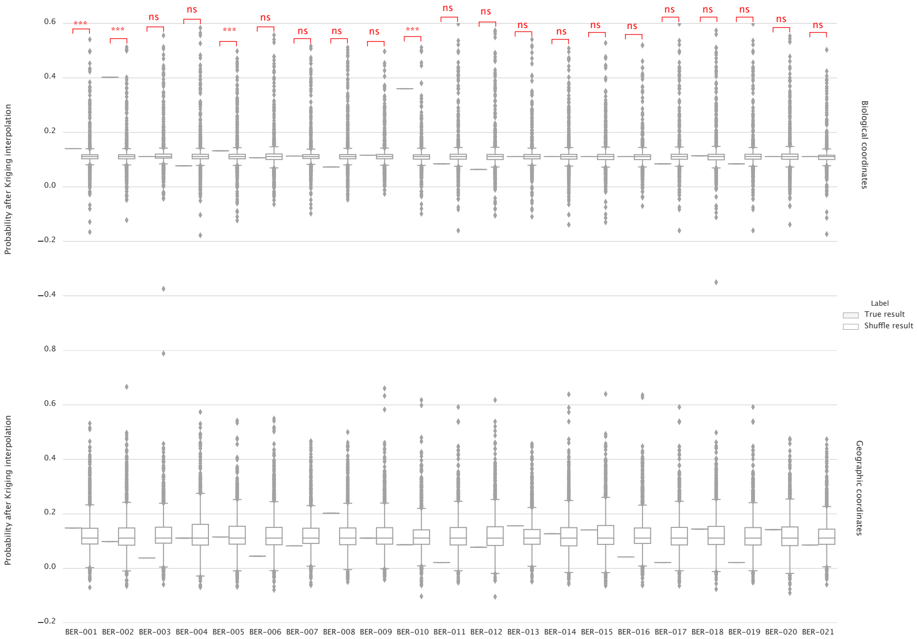

Figure S1: Estimated probabilities of samples originated from Berlin. The probabilities were interpolated from the other 9 European cities except Berlin using the proposed framework. Kriging interpolation is performed on the biological coordinates. For each sample, the probability on the left side is resulted from original training data and those on the right side are from permuted training samples.

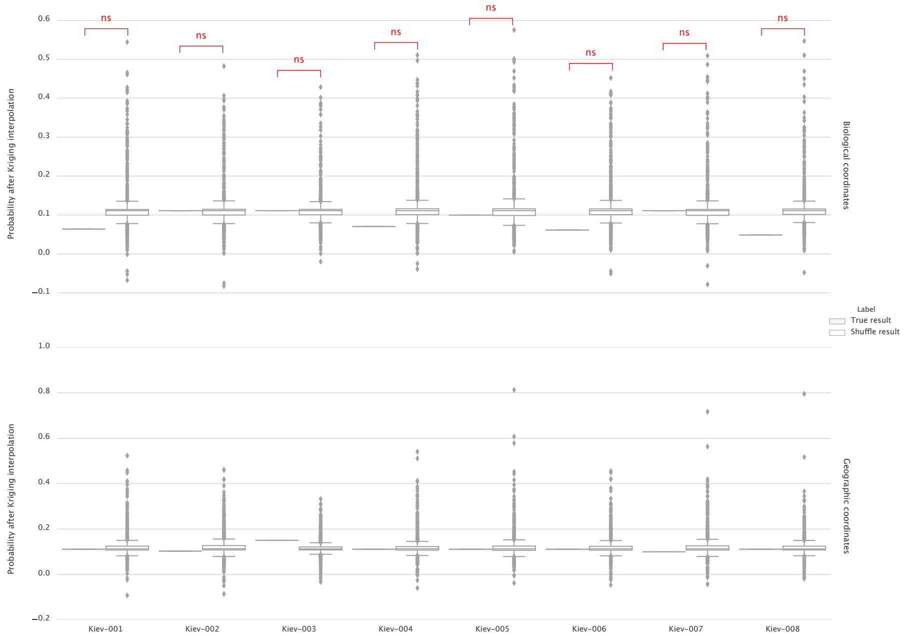

Figure S2: Estimated probabilities of samples originated from Kiev. The probabilities were interpolated from the other 9 European cities except Kiev using the proposed framework. Kriging interpolation is performed on the biological coordinates. For each sample, the probability on the left side is resulted from original training data and those on the right side are from permuted training samples.

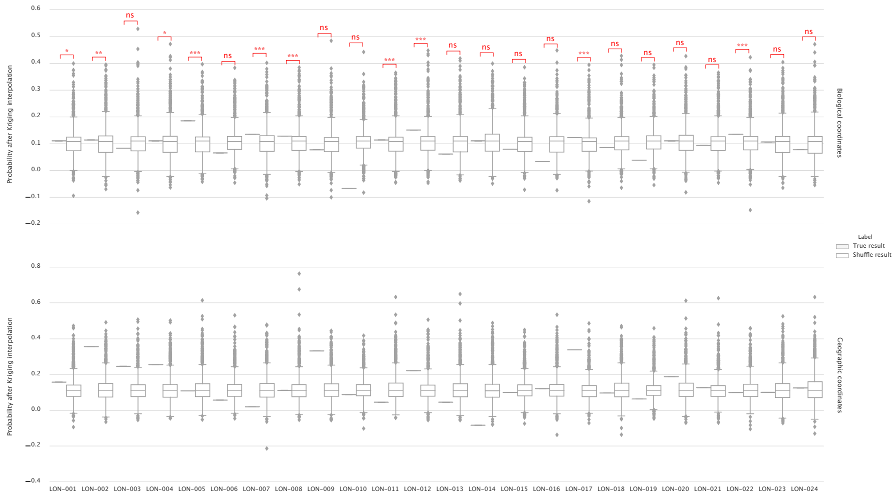

Figure S3: Estimated probabilities of samples originated from Kiev. The probabilities were interpolated from the other 9 European cities except Kiev using the proposed framework. Kriging interpolation is performed on the biological coordinates. For each sample, the probability on the left side is resulted from original training data and those on the right side are from permuted training samples.

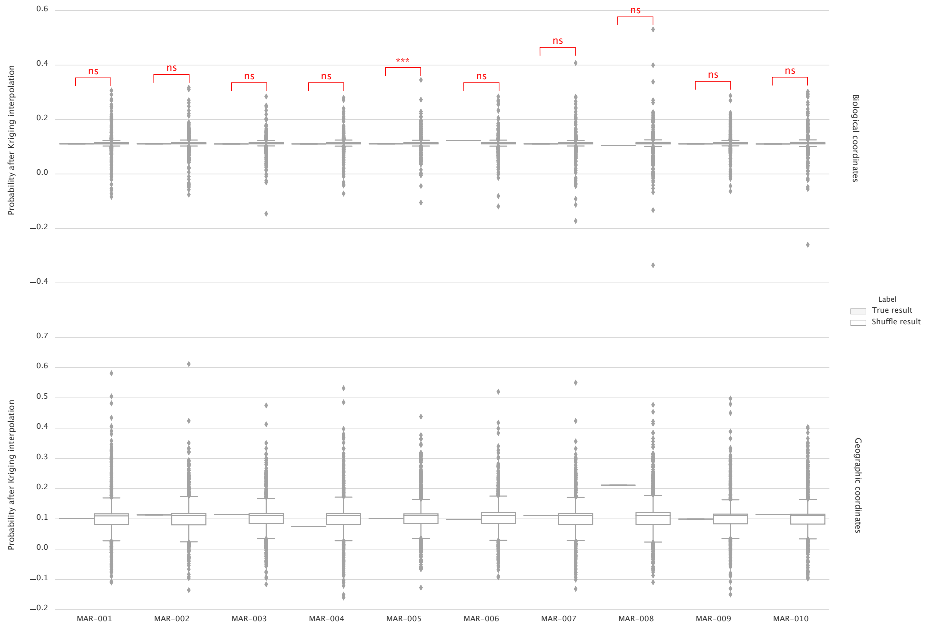

Figure S4: Estimated probabilities of samples originated from Marseille. The probabilities were interpolated from the other 9 European cities except Marseille using the proposed framework. Kriging interpolation is performed on the biological coordinates. For each sample, the probability on the left side is resulted from original training data and those on the right side are from permuted training samples.

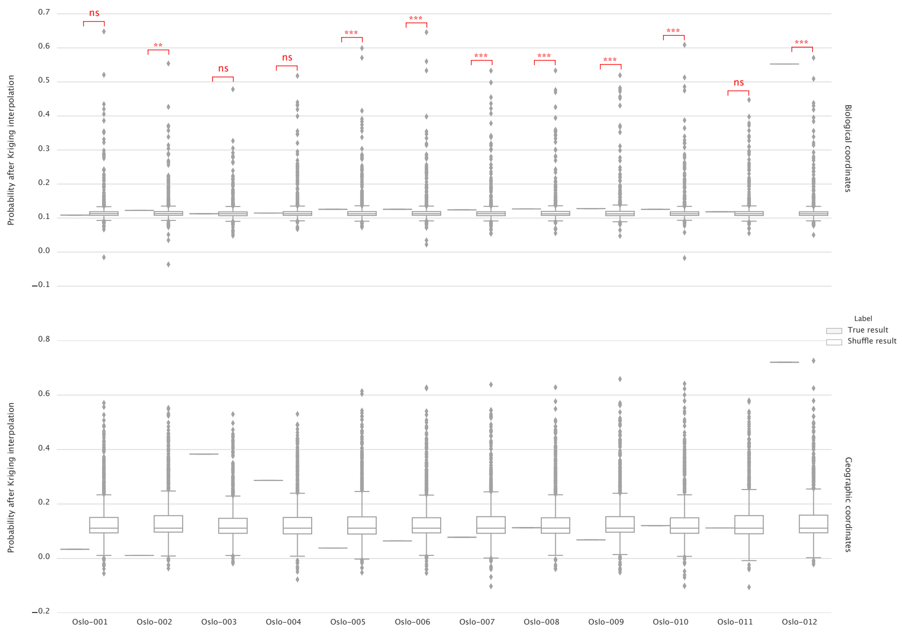

Figure S5: Estimated probabilities of samples originated from Oslo. The probabilities were interpolated from the other 9 European cities except Oslo using the proposed framework. Kriging interpolation is performed on the biological coordinates. For each sample, the probability on the left side is resulted from original training data and those on the right side are from permuted training samples.

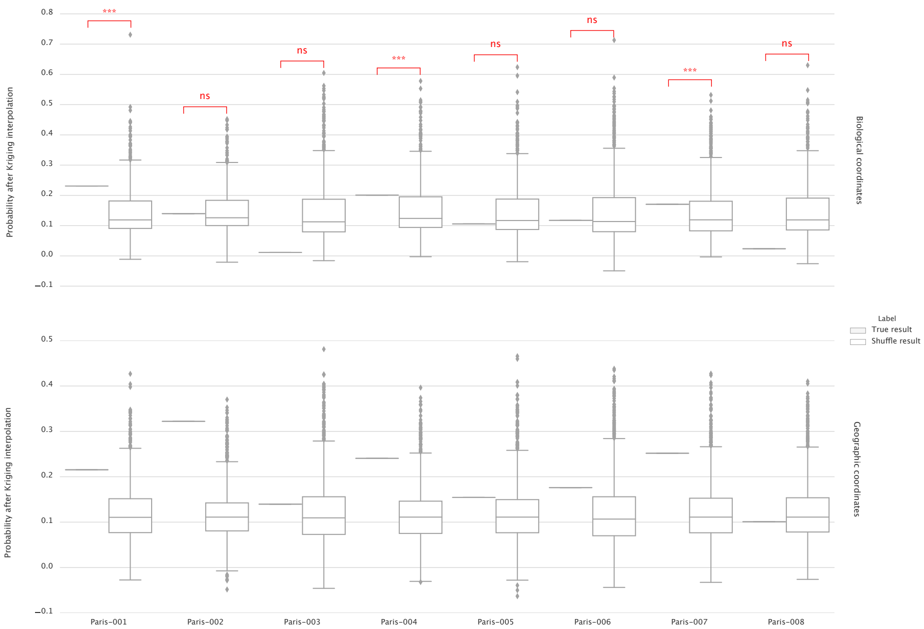

Figure S6: Estimated probabilities of samples originated from Paris. The probabilities were interpolated from the other 9 European cities except Paris using the proposed framework. Kriging interpolation is performed on the biological coordinates. For each sample, the probability on the left side is resulted from original training data and those on the right side are from permuted training samples.

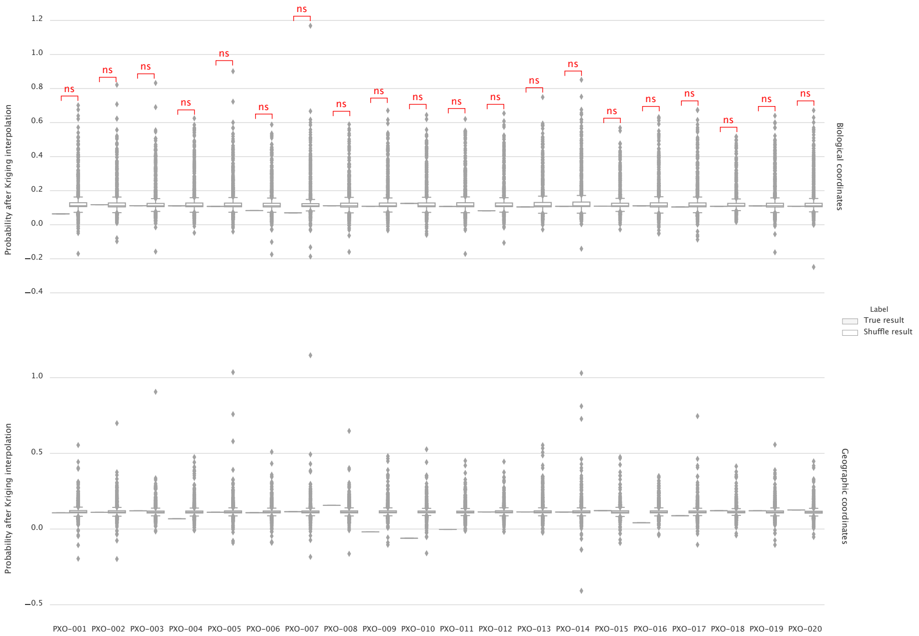

Figure S7: Estimated probabilities of samples originated from Porto. The probabilities were interpolated from the other 9 European cities except Porto using the proposed framework. Kriging interpolation is performed on the biological coordinates. For each sample, the probability on the left side is resulted from original training data and those on the right side are from permuted training samples.

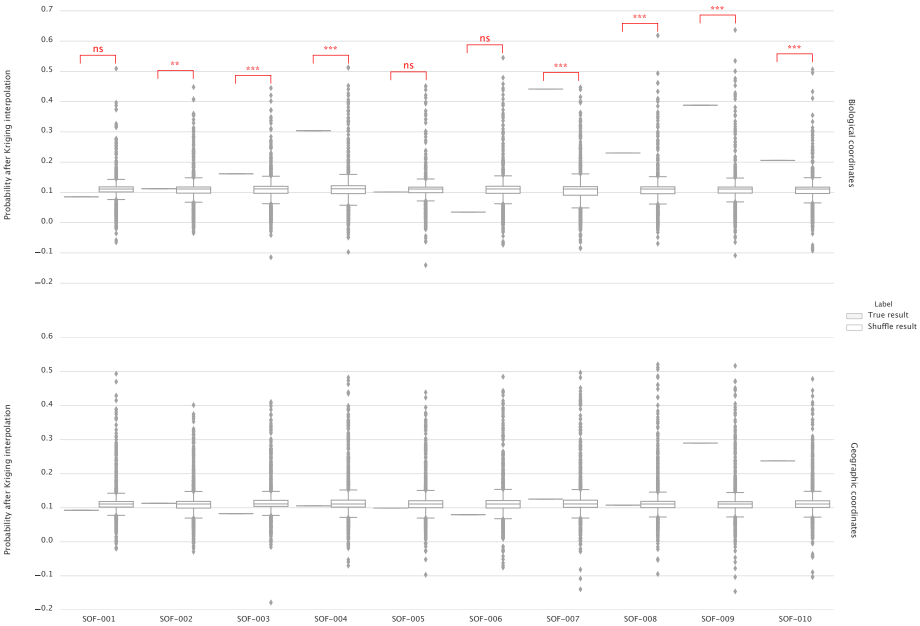

Figure S8: Estimated probabilities of samples originated from Sofia. The probabilities were interpolated from the other 9 European cities except Sofia using the proposed framework. Kriging interpolation is performed on the biological coordinates. For each sample, the probability on the left side is resulted from original training data and those on the right side are from permuted training samples.

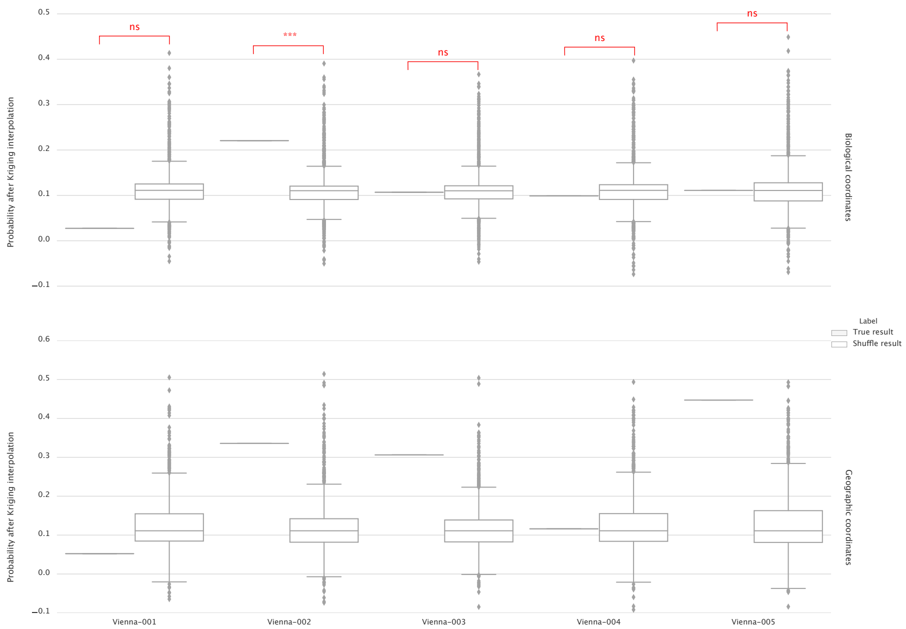

Figure S9: Estimated probabilities of samples originated from Vienna. The probabilities were interpolated from the other 9 European cities except Vienna using the proposed framework. Kriging interpolation is performed on the biological coordinates. For each sample, the probability on the left side is resulted from original training data and those on the right side are from permuted training samples.
